# Prevalence and incidence of left ventricular systolic dysfunction and adverse outcomes in patients receiving *de novo* and replacement pacemaker therapy for bradycardia

**DOI:** 10.1093/ehjopen/oeag069

**Published:** 2026-04-27

**Authors:** Nurul H Abdul Samad, John Gierula, Judith E Lowry, Sam Straw, Samuel D Relton, Stephe Kamalathasan, Richard M Cubbon, Mark T Kearney, Klaus K Witte, Maria F Paton

**Affiliations:** Leeds Institute of Cardiovascular and Metabolic Medicine, Multidisciplinary Cardiovascular Research Centre, University of Leeds, Cardiovascular Research Facility, F Floor, Jubilee Wing, Leeds General Infirmary, LS1 3EX, Clarendon Way, Leeds LS2 9JT, UK; Leeds Institute of Cardiovascular and Metabolic Medicine, Multidisciplinary Cardiovascular Research Centre, University of Leeds, Cardiovascular Research Facility, F Floor, Jubilee Wing, Leeds General Infirmary, LS1 3EX, Clarendon Way, Leeds LS2 9JT, UK; Leeds Institute of Cardiovascular and Metabolic Medicine, Multidisciplinary Cardiovascular Research Centre, University of Leeds, Cardiovascular Research Facility, F Floor, Jubilee Wing, Leeds General Infirmary, LS1 3EX, Clarendon Way, Leeds LS2 9JT, UK; Leeds Institute of Cardiovascular and Metabolic Medicine, Multidisciplinary Cardiovascular Research Centre, University of Leeds, Cardiovascular Research Facility, F Floor, Jubilee Wing, Leeds General Infirmary, LS1 3EX, Clarendon Way, Leeds LS2 9JT, UK; Leeds Institute of Health Sciences, University of Leeds, Clarendon Way, Leeds LS2 9JT, UK; Leeds Institute of Cardiovascular and Metabolic Medicine, Multidisciplinary Cardiovascular Research Centre, University of Leeds, Cardiovascular Research Facility, F Floor, Jubilee Wing, Leeds General Infirmary, LS1 3EX, Clarendon Way, Leeds LS2 9JT, UK; Leeds Institute of Cardiovascular and Metabolic Medicine, Multidisciplinary Cardiovascular Research Centre, University of Leeds, Cardiovascular Research Facility, F Floor, Jubilee Wing, Leeds General Infirmary, LS1 3EX, Clarendon Way, Leeds LS2 9JT, UK; Leeds Institute of Cardiovascular and Metabolic Medicine, Multidisciplinary Cardiovascular Research Centre, University of Leeds, Cardiovascular Research Facility, F Floor, Jubilee Wing, Leeds General Infirmary, LS1 3EX, Clarendon Way, Leeds LS2 9JT, UK; Leeds Institute of Cardiovascular and Metabolic Medicine, Multidisciplinary Cardiovascular Research Centre, University of Leeds, Cardiovascular Research Facility, F Floor, Jubilee Wing, Leeds General Infirmary, LS1 3EX, Clarendon Way, Leeds LS2 9JT, UK; Leeds Institute of Cardiovascular and Metabolic Medicine, Multidisciplinary Cardiovascular Research Centre, University of Leeds, Cardiovascular Research Facility, F Floor, Jubilee Wing, Leeds General Infirmary, LS1 3EX, Clarendon Way, Leeds LS2 9JT, UK

**Keywords:** Heart failure, Pacemaker, Mortality, Left ventricular systolic dysfunction

## Abstract

**Aims:**

Right ventricular pacing (RVP) is associated with left ventricular systolic dysfunction (LVSD) and heart failure (HF). We investigated the prevalence and predictors of LVSD and adverse HF outcomes in patients undergoing *de novo* or replacement RVP for bradycardia.

**Methods and results:**

Prospective data were collected from 1024 patients receiving *de novo* (2014–2017, *n* = 514) or generator replacement (2008–2011, *n* = 510) RVP at a UK tertiary centre. Logistic regression models were used to identify predictors of LVSD, defined as left ventricular ejection fraction (LVEF) < 50%, and subsequent HF hospitalization (HFH) and all-cause mortality. Overall, 61% were male; mean age 76 ± 11 years. *De novo* patients were more often male (66% vs. 56%) and more likely to have ischaemic heart disease (IHD; 31% vs. 14%) and diabetes (24% vs. 6%), but less likely to have atrial fibrillation (AF; 25% vs. 33%) than replacement patients (all *P* < 0.01). LVSD was present in 344 (37%) and was more frequent in *de novo* than replacement cases (20% vs. 17%, *P* < 0.01). Independent predictors of LVSD were IHD [odds ratio (OR) = 2.56, confidence interval (CI): 1.58–4.13] and ventricular pacing burden >80% (OR = 2.13, CI: 1.29–3.52). Over a median 30 (IQR: 16–42) months, 341 (33%) experienced HFH or death, more commonly after replacement than *de novo* RVP (25% vs. 8%, *P* < 0.01). Predictors of adverse outcomes included replacement status [hazard ratio (HR) = 2.27, CI: 1.78–4.16], older age, AF, and LVEF <50%.

**Conclusion:**

LVSD is common in RVP recipients and largely driven by comorbidities rather than pacing burden. Screening and targeted therapy in high-risk patients may improve outcomes and optimize resource use.

## Graphical Abstract

**Graphical Abstract:**
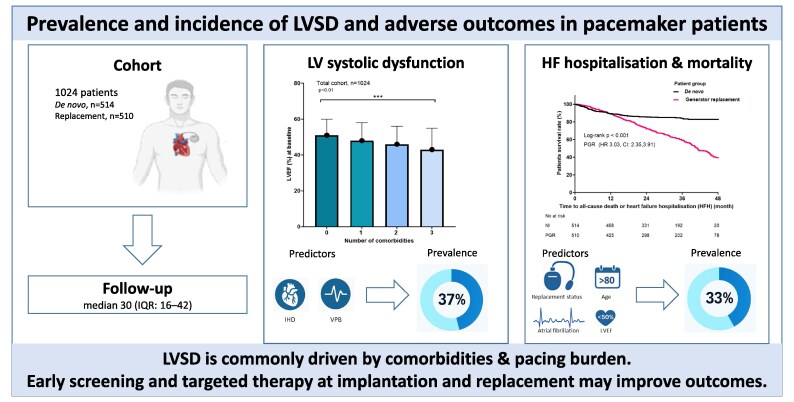

What’s known?

- High ventricular pacing burden has been associated with left ventricular systolic dysfunction and adverse cardiovascular clinical outcomes

**What’s new?**

- *De novo* and generator replacement implantation are important time points for considering cardiac device and medical therapy optimization.
- Ventricular pacing burden remains a key risk factor for prevalent left ventricular dysfunction, even in the era of modern devices with right ventricular pacing avoidance algorithms.
- Comorbid patients, particularly with a history of ischaemic heart disease, atrial fibrillation, and a high ventricular pacing requirement could be a target population in which to consider screening for left ventricular systolic dysfunction.

## Introduction

Pacemaker implantation is the only long-term treatment for bradycardia and is proven to extend longevity and improve quality of life.^1^ Approximately 400 000 people in European Society of Cardiology (ESC) countries undergo pacemaker implantation each year.^2^ The right ventricular apical position has been the standard of care for more than half a century and is proven to be safe and reliable.^3,4^ However, standard right ventricular pacing (RVP) can cause a dyssynchronous left ventricular (LV) contraction pattern and has been associated with adverse LV remodelling, which is associated with the clinical syndrome of heart failure (HF).^5,6^

Recently, alternate RV lead positions have been proposed as potential options to reduce the incidence of LV systolic dysfunction (LVSD) and adverse HF outcomes in people needing treatment for bradycardia. However, these ‘conduction systems pacing’ (CSP) approaches are associated with unique challenges including a long learning curve that currently limits their widespread adoption.^7–10^ Moreover, whilst short-term observational data suggest that CSP may be associated with fewer adverse HF outcomes than RVP, there are limited long-term data.^11–14^ Hence, most pacemaker implants worldwide continue to utilize standard RVP. In addition, there are millions of patients with legacy devices utilizing apical RVP.

Therefore, this study aimed to evaluate the prevalence of LVSD and describe patient-oriented clinical and diagnostic predictors of LVSD and adverse clinical outcomes in patients undergoing *de novo* or replacement pacemaker implantation for bradycardia.

## Methods

This observational study was based on two historically collected prospective cohorts, with consecutive patients enrolled under contemporaneous ethical approval, a pre-specified 12-month follow-up, and prospectively recorded clinical, echocardiographic, and outcome data at a single tertiary hospital in the UK. Systematic protocoled screening for LVSD prior to pacemaker implantation was not mandated within the study framework.

## Study population

All patients undergoing either *de novo* RVP implantation for ESC guideline indications^15^ between 2014 and 2017 or a RVP generator replacement between 2008 and 2011 in a single tertiary centre in the UK, aged ≥18 years, and capable of providing informed written consent were invited to participate. Device therapy was delivered according to prevailing UK clinical guidelines. Exclusion criteria were limited to cognitive impairment preventing the ability to provide informed consent, or the previous or planned receipt of implantable cardioverter defibrillator or cardiac resynchronization therapy (CRT) devices.

## Data collection

Demographic and clinical data, including age, sex, vital signs, height, weight, comorbidities, medical history, current medical therapy, and digital echocardiography images, were collected at baseline assessment. Echocardiography was conducted according to the British Society of Echocardiography guidelines for a minimum dataset which included assessment of left ventricular (LV) function, LV and left atrial (LA) size.^16^ Pacemaker interrogation collected programmed pacing mode, base rate, and cumulative pacing percentages for atrium and ventricle. Patients receiving *de novo* implantation were offered repeated transthoracic echocardiography and device interrogation at a minimum of 12 months follow-up. Digital follow-up of all patients using NHS systems permitted assessment of clinical outcomes.

## Outcome measures

The primary outcome measure was the prevalence of LVSD, defined as an LV ejection fraction (LVEF) less than 50%, consistent with prior echocardiographic studies evaluating mechanical dyssynchrony, and in line with guideline definitions of mildly reduced LV systolic function.^15^ This study was not designed to diagnose pacing-induced cardiomyopathy; rather, LVSD was assessed as a functional outcome using paired echocardiography to evaluate early changes in LV systolic function following pacemaker implantation. Secondary outcomes included identifying clinical features associated with the presence of LVSD and the composite of all-cause mortality or HF hospitalization (HFH). HFH was defined as an unplanned hospital admission of ≥24 h with a primary diagnosis of HF, accompanied by new or worsening symptoms and signs requiring intravenous diuretic therapy, vasoactive treatment, or escalation of guideline-directed heart failure therapy. HFH events were ascertained using electronic health record review, hospital episode coding, and structured digital follow-up, with events identified through digital follow-up confirmed by contemporaneous discharge documentation or verified admission records. All HFH events underwent clinician adjudication to ensure consistency with predefined criteria and to exclude non-HF related admissions.

Exploratory secondary analyses were performed to assess the risk of a clinically significant reduction in LVEF in patients receiving *de novo* implantation,^17^ defined as a reduction of equal to or more than 10% from baseline, associated with baseline ventricular pacing burden (VPB) and comorbidity burden. The comorbidity score was calculated based on the presence of ischaemic heart disease (IHD), diabetes mellitus (DM), and atrial fibrillation (AF), with patients stratified into groups with 0, 1, 2, or 3 comorbidities.

## Statistical analysis

Continuous data were firstly assessed for normality using the Shapiro-Wilk test. Normally distributed data are presented as mean ± standard deviation, continuous non-normally distributed data as median (interquartile range), and categorical data are presented as number (percentage). Student *t*-tests or analysis of variance were used to analyse normally distributed continuous data, Mann–Whitney *U*-tests or Kruskal–Wallis *H*-tests for non-normally distributed continuous data, and Pearson χ^2^ tests for categorical data.

Logistic regression analysis was undertaken to investigate risk factors for prevalent LVSD. We prespecified the inclusion of seven key clinical variables based upon previous studies,^6,18–21^ which were: Patient group (*de novo* or replacement), male sex, age, history of IHD, type 2 diabetes mellitus, the presence of AF, and the VPB (<40%, 40–80%, or >80%), in univariable and multivariable regression analysis. Bootstrap analysis was applied to assess model robustness. We estimated that we would require approximately 7 events per covariate to prevent overfitting, therefore at least 70 observed events were required to provide adequate power in the regression models.^22–24^ Subsequently, the sensitivity and specificity of key clinical variables and number of comorbidities as predictors of LVSD were analysed.

Time to the composite outcome of all-cause mortality or HFH was analysed using Kaplan–Meier survival curves, with patients stratified according to baseline VPB (<40%, 40–80%, and >80%). Given recent data suggesting the potential benefit of CRT upgrade in patients with lower VPB than previously studied, a secondary exploratory analysis was conducted to specifically examine a subgroup with VPB >20%.^25^

Predictors of the composite outcome of all-cause mortality or HFH were assessed using Cox proportional hazards models. Clinical predictor variables assessed were those described above with the addition of LVEF, LV end-systolic diameter (LVESD) and LV end-diastolic diameter (LVEDD).

All statistical analyses were performed using IBM SPSS Statistics (version 27.0; IBM Corp., Armonk, NY, USA, 2020). Figures, including Kaplan–Meier survival curves with numbers at risk and forest plots, were generated using GraphPad Prism (version 10.2.0; GraphPad Software, San Diego, CA, USA, 2024) for graphical presentation. Statistical significance was pre-specified at *P* < 0.05, accompanied by a 95% confidence interval (CI).

## Ethics and governance

The sponsor of the study was the University of Leeds and ethical approval was obtained for both cohorts in advance of any patient-related activity (12/YH/0487 and 08/H1307/12, respectively). All patients provided informed written consent and all research activity complied with the principles of the Declaration of Helsinki.

## Results

### Population characteristics

A total of 1024 patients were prospectively enrolled, of whom 514 patients were enrolled shortly after *de novo* implantation and 510 after a generator replacement. Baseline characteristics of the entire cohort, and by group are shown in *Table 1*. The mean age of patients undergoing *de novo* and generator replacement were similar (76 ± 10 years vs. 76 ± 12 years, *P* = 0.33). Compared with those receiving replacement, *de novo* patients were more likely to be male (66% vs. 56%, *P* < 0.01), have a history of IHD (31% vs. 14%, *P* < 0.01), and to have type 2 diabetes mellitus (24% vs. 6%, *P* < 0.01), but less likely to have AF (25% vs. 33%, *P* < 0.01). Patients in the replacement group compared to the *de novo* group, were more likely to have no comorbidities (59% vs. 43%), and less likely to have two (7% vs. 18%) or three (1% vs. 3%) comorbidities (*P* < 0.01).

**Table 1:** Baseline characteristics of the study cohort

	Total cohort *n* = *1024*	NI group *n* = *514*	PGR group *n* = *510*	*P*-value
**Age (years)**	76 (±11)	76 (±10)	76 (±12)	0.33
**Sex (male)**	628 [61%]	341 [66%]	287 [56%]	<0.01*
**Height (cm)**	167 (±12)	167 (±10)	166 (±16)	0.66
**Weight (kg)**	78 (±18)	79 (±18)	76 (±18)	0.01*
**Atrial fibrillation**	296 [29%]	128 [25%]	168 [33%]	<0.01*
**Type II Diabetes Mellitus**	153 [15%]	121 [24%]	32 [6%]	<0.01*
**Ischaemic heart diseases**	232 [23%]	160 [31%]	72 [14%]	<0.01*
MI	154 [15%]	119 [23%]	35 [7%]	<0.01*
PCI	63 [6%]	46 [9%]	17 [3%]	<0.01*
CABG	113 [11%]	58 [11%]	55 [11%]	<0.01*
**Baseline pacing indication**				
Sinus node disease	385 [40%]	159 [31%]	227[45%]	<0.01*
Atrioventricular block	367 [38%]	218 [42%]	179 [35%]	<0.01*
Other	222 [23%]	137 [27%]	104 [20%]	<0.01*
**Medical therapy**				
β-blockers	320 [31%]	214 [42%]	106 [21%]	<0.01*
ACE-inhibitors	347 [34%]	236 [46%]	111 [22%]	<0.01*
Spironolactone	126 [15%]	110 [21%]	16 [3%]	0.01*
Furosemide	178 [17%]	110 [21%]	68 [13%]	<0.01*
**Pacing system**				
Dual chamber pacing	810 [79%]	400 [78%]	410 [80%]	0.31
**Pacing programming**				
DDD (R)	490 [48%]	172 [34%]	217 [44%]	<0.01*
RV pacing avoidance algorithm	193 [19%]	193 [38%]	79 [16%]	<0.01*
VVI (R)	249 [24%]	116 [23%]	143 [29%]	<0.01*
AAI (R)	27 [3%]	25 [5%]	3 [1%]	<0.01*
DDI (R)	46 [4%]	5 [1%]	34 [7%]	<0.01*
VDD	26 [2%]	3 [1%]	23 [5%]	<0.01*
**Pacing requirement**				
Rate response	321 [31%]	78[15%]	215 [42%]	<0.01*
Base rate (bpm)	54 (±8)	50 (±4)	58 (±9)	<0.01*
Max track rate (bpm)	126 (±12)	130 (±11)	123 (±12)	<0.01*
APB (%)	9 (1–52)	2 (1–25)	39 (3–85)	<0.01*
VPB (%)	14 (1–96)	10 (1–83)	24 (1–99)	<0.01*
**Echocardiographic measurement**				
LVEF (%)	50 (±10)	50 (±8)	50 (±12)	0.61
LVEDD (mm)	47 (±7)	47 (±6)	47 (±7)	0.83
LVESD (mm)	35 (±7)	35 (±7)	35 (±8)	0.31
LA diameter (mm)	41 (±8)	41 (±7)	41 (±9)	0.82

Continuous data are presented as mean (± standard deviation) or median (interquartile range) and categorical data are presented as *n* (%). A *P*-value ≤0.05 denotes * was considered significant.

MI; myocardial infarction, PCI; percutaneous coronary intervention, CABG; coronary artery bypass graft, ACE inhibitors; angiotensin-converting enzyme inhibitor, β-blockers; beta-blockers, RV; right ventricular, APB; atrial pacing burden, VPB; ventricular pacing burden, LVEF; left ventricular ejection fraction, LVESD; left ventricular end-systolic dysfunction, LVEDD; left ventricular end-diastolic diameter, LA; left atrial.

### Pacing indication, device prescription and requirement

Patients receiving *de novo* devices were more likely to have a pacing indication for atrioventricular block (AVB) compared to those undergoing generator replacement (42% vs. 35%, *P* < 0.01). However, device prescription was overwhelmingly dual chamber in both groups with no difference between them (78% vs. 80%, *P* = 0.31, respectively). Patients receiving *de novo* devices were more likely than those receiving a generator replacement to have RVP avoidance algorithms activated (38% vs. 16%, *P* < 0.01), less likely to receive rate-adaptive pacing (15% vs. 42%, *P* < 0.01), and demonstrated a lower requirement for pacing in both atrium [2% (IQR:1–25) vs. 39% (3–85), *P* < 0.01] and ventricle [10% (1–83) vs. 24% (1–99), *P* < 0.01].

### Medication prescription

Cardiovascular medical therapy was more commonly prescribed in the *de novo* group compared with those undergoing generator replacement, including β-blockers (42% vs. 21%, *P* < 0.01), angiotensin converting enzyme inhibitors (46% vs. 22%, *P* < 0.01), mineralocorticoid receptor antagonists (21% vs. 3%, *P* < 0.01), and loop diuretics (21% vs. 13%, *P* < 0.01).

### Factors associated with left ventricular systolic dysfunction

Mean LV dimensions and LVEF at baseline were not significantly different between patient groups (LVEDD 47 ± 6 mm vs. 47 ± 7 mm, *P* = 0.83; LVESD 35 ± 7 mm vs. 35 ± 8 mm, *P* = 0.31, and LVEF 50 ± 8% vs. 50 ± 12%, *P* = 0.61).

Among the entire cohort, 344 patients (37%) had LV systolic dysfunction (EF <50%). LVSD was present in 182 of 514 *de novo* implantations (35%) and in 162 of 510 generator replacements (32%) (*P* = 0.21). Univariable analysis revealed that male sex [Odds Ratio (OR) = 2.19, CI:1.66,2.91, *P* < 0.01], age > 80 years (OR = 1.64, CI:1.04, 2.57, *P* = 0.03), a history of IHD (OR = 2.49, CI:1.80,3.44, *P* < 0.01), the presence of AF (OR = 1.36, CI:1.03,1.81, *P* = 0.03) and a VPB >80% (OR = 2.34, CI:1.55,3.52, *P* < 0.01) were independently associated with LVSD (see Supplementary material online, *Table S1*).

**Figure 1:**
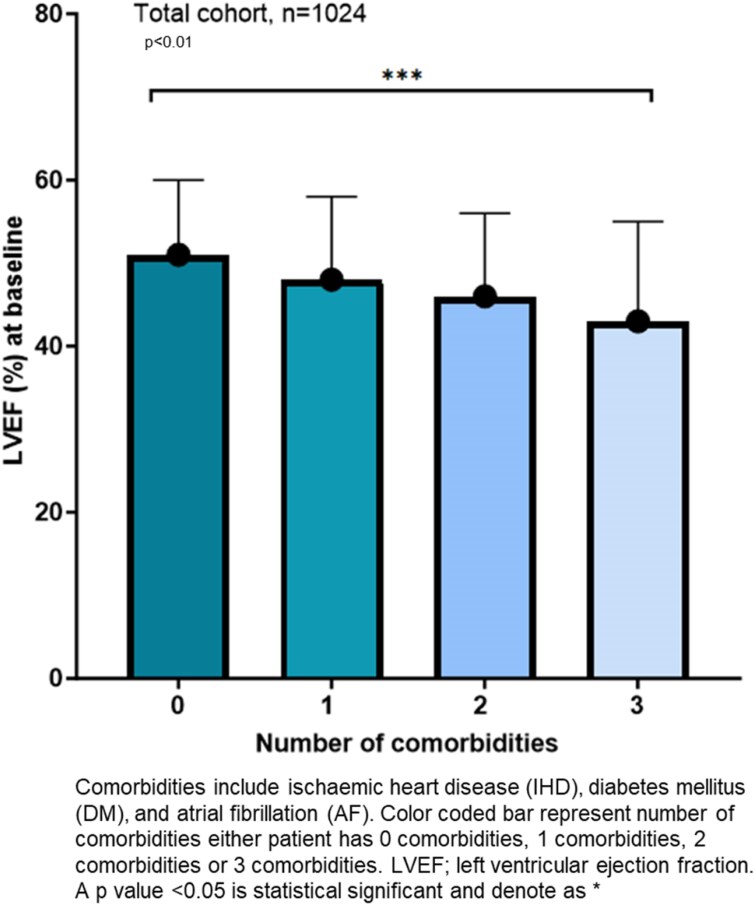
Left ventricular ejection fraction (LVEF) percentages according to number of comorbidities at baseline in the total cohort (*n* = 1024).

LVEF, however, was significantly lower in those with increasing numbers of comorbidities (IHD, DM and AF) (51 ± 9% for 0 comorbidities; 48 ± 10% for 1 comorbidity; 46 ± 10% for 2 comorbidities; 43 ± 12% for 3 comorbidities, *P* < 0.01) (*Figure 1*).

**Figure 2:**
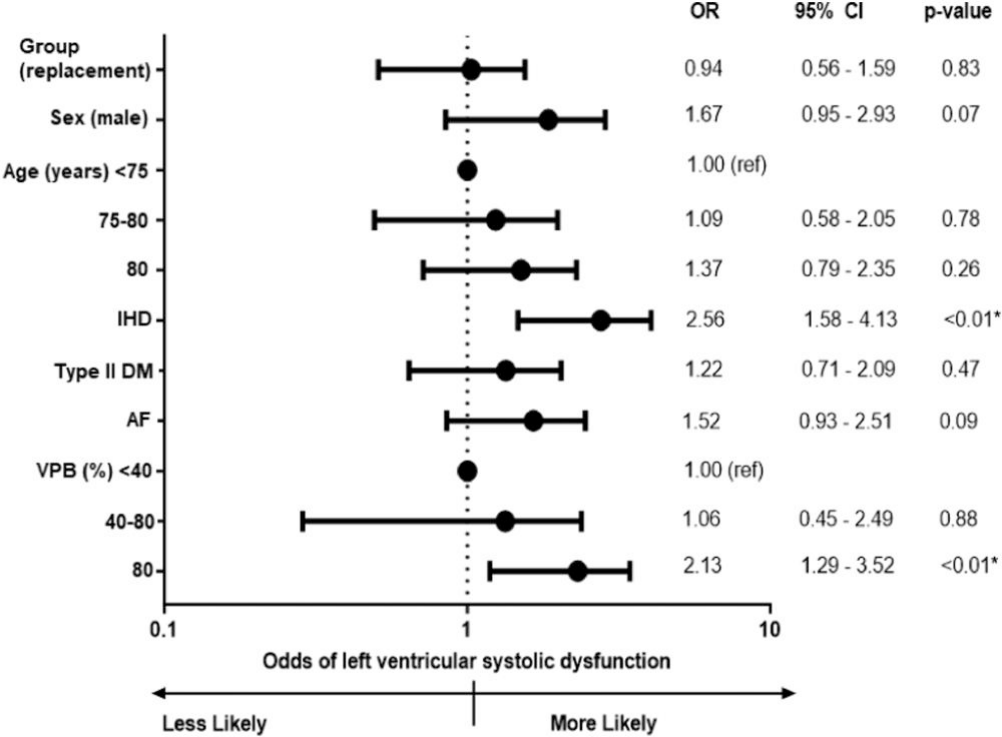
Factors associated with left ventricular dysfunction amongst pacemaker’s patients. AF, atrial fibrillation; DM, diabetes mellitus; IHD, ischaemic heart disease; VPB, ventricular pacing burden. A *P* value <0.05 is statistical significance and denote as *.

In a multivariable analysis of clinically and statistically relevant variables, having a history of IHD (OR = 2.56, CI: 1.58, 4.13, *P* < 0.01), and VPB >80% (OR = 2.13, CI: 1.29, 3.52, *P* = 0.01) were independently associated with LVSD (*Figure 2*). Sensitivity and specificity for prevalent LVSD in those with IHD and VPB >80 was 77% and 53%, respectively.

### Change in LVEF following *de novo* right ventricular pacemaker implantation

**Figure 3:**
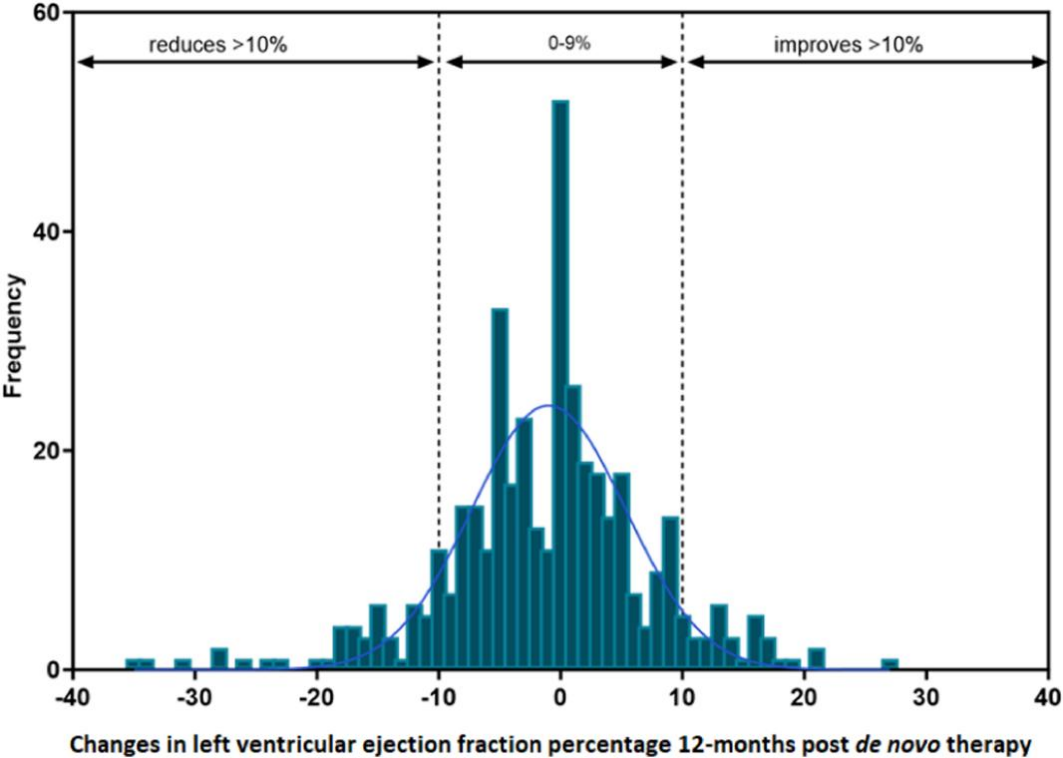
Changes in left ventricular ejection fraction percentage at 12- month post procedure in *de novo* patients (*n* = 514).

**Figure 4:**
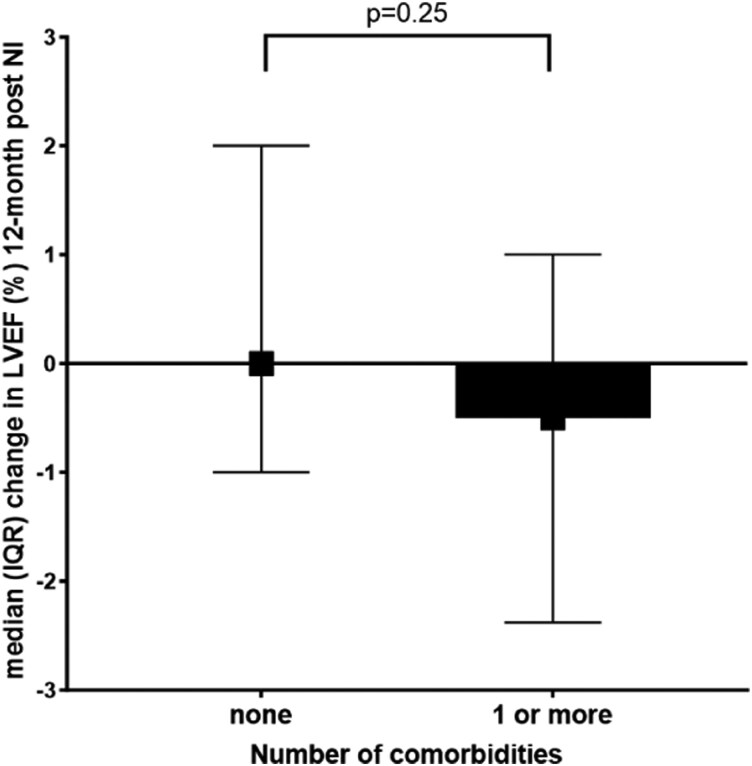
Median (IQR) change in LVEF percentage according to number of comorbidities in *de novo* patients (*n* = 514). Comorbidities include ischaemic heart disease (IHD), diabetes mellitus (DM), and atrial fibrillation (AF). Bar represents number of comorbidities either patient has 0 comorbidities or 1 or more comorbidities. LVEF; left ventricular ejection fraction. *P* = 0.25.

In the first 12 months following *de novo* pacemaker implantation, median change in LVEF was −1.00 (IQR: −8 to 6)% (*Figure 3*). A total of 31 (8%) patients experienced a clinically significant reduction in LVEF, defined as a reduction of equal to or more than 10%.^17^ Median LVEF change was not significantly associated with the number of comorbidities in the first 12 months following *de novo* implantation (*P* = 0.25) (*Figure 4*).

### Factors associated with event-free to first composite outcome of all-cause death or HFH

After a median follow-up period of 30^16–42^ months, a total of 341 (33%) patients had been hospitalized for HF or had died, of which 207 (20%) patients experienced a first event of HFH, and 134 (13%) patients had a primary event of death. Of these, 79 (8%) were *de novo* patients, and 262 (25%) were patients that had undergone generator replacement (*P* = 0.02). When stratified by group, 62 (12%) *de novo* patients experienced HFH first and 17 (3%) died first, compared with 145 (28%) and 117 (23%), respectively, in the replacement group (*P* < 0.01). In univariable analyses, receiving a generator replacement [Hazard Ratio (HR) = 3.03, CI: 2.35,3.96; *P* = 0.02], being older than 80 years (HR = 2.41, CI:1.51,3.84; *P* < 0.01), the presence of AF (HR = 2.25, CI:1.73,2.94; *P* < 0.01), requiring >80% ventricular pacing (HR = 2.14, CI:1.21,3.76; *P* = 0.01), and LVEF <40% (HR = 0.50, CI:0.28,0.87; *P* = 0.02) were independently associated with time to first composite outcome of HFH or all-cause death (see Supplementary material online, *Table S2*).

**Figure 5:**
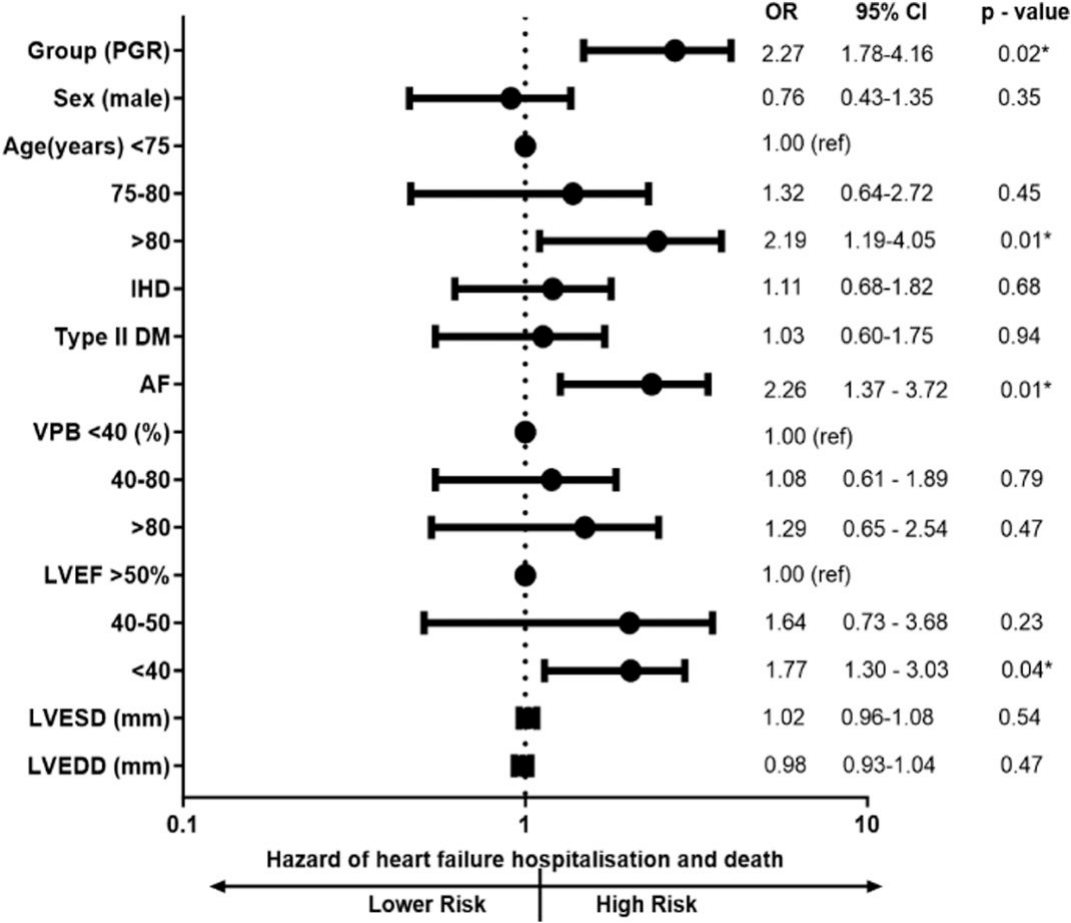
Predictors of time to composite outcome of all-cause death or heart failure hospitalization (HFH) amongst pacemaker patients after a minimum of 12 months follow up with a censor date on 28 July 2018 for *de novo* patients vs. 31 December 2012 for generator replacement patients. AF, atrial fibrillation; DM, diabetes mellitus; IHD, ischaemic heart disease; LVEF, left ventricular ejection fraction; LVESD, left ventricular end systolic diameter; LVEDD, left ventricular end diastolic diameter; VPB, ventricular pacing burden. A *P* value <0.05 is statistical significance and denote as *.

**Figure 6:**
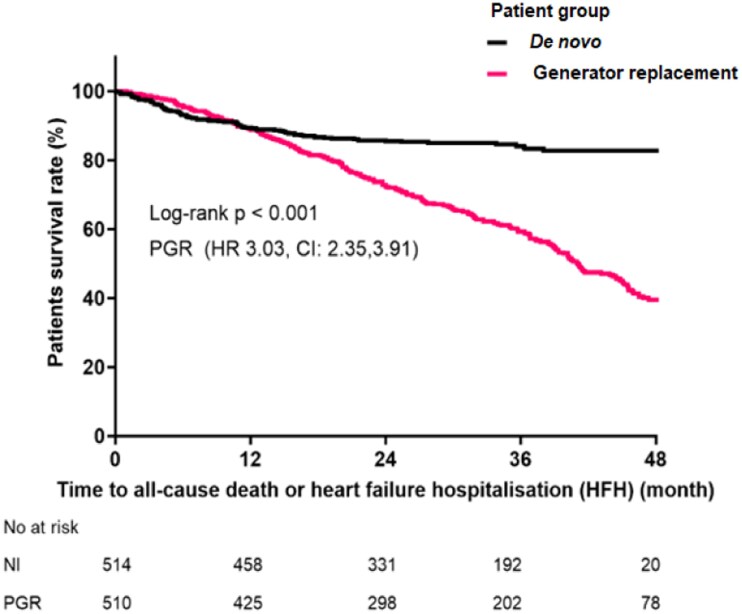
Kaplan–Meier analysis showing estimates free from the composite outcome of all-cause death or heart failure hospitalization (HFH) according to patient group (*de novo*, generator replacement) (log rank *P* < 0.01).

After accounting for clinically relevant variables in a multivariable model, receiving a generator replacement (HR = 2.27, CI:1.78,4.16, *P* = 0.02), being older than 80 years (HR = 2.19, CI:1.19,4.05, *P* < 0.01), having a history of AF (HR = 2.26, CI:1.37,3.72, *P* < 0.01) and having an LVEF <40% (HR = 1.77, CI:1.30,3.03, *P* = 0.04) remained independently associated with earlier occurrence of the composite outcome of HF or all-cause death (*Figures 5* and *6*).

**Figure 7:**
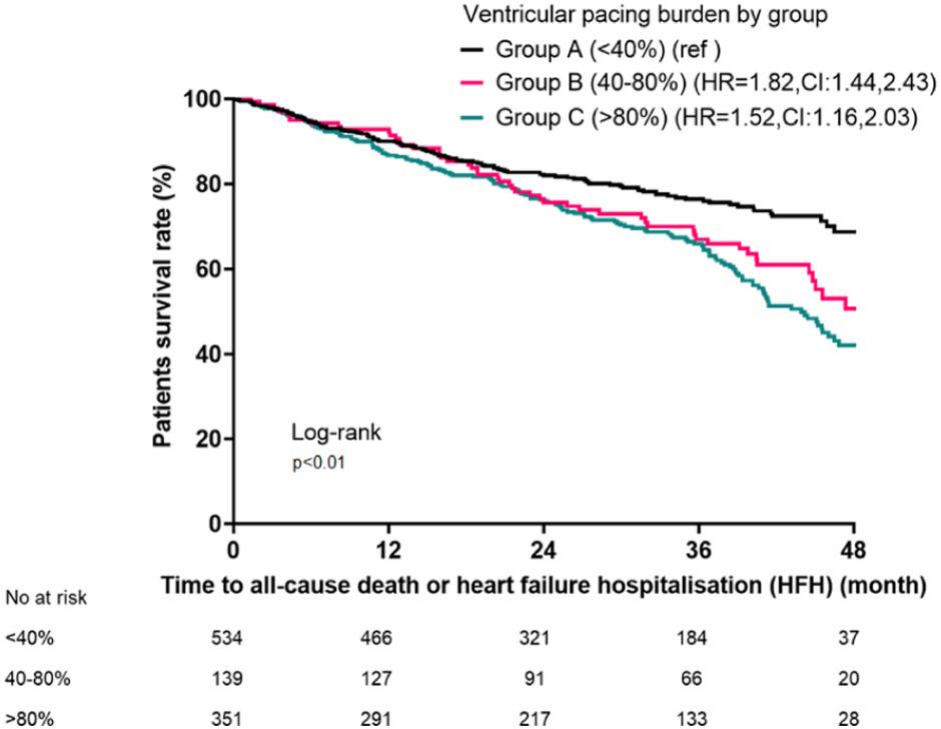
Kaplan–Meier showing estimates patients survival rate (group: from composite outcome of all-cause death or heart failure hospitalization (HFH) or by ventricular pacing burden (VPB) group: group a < 40% as reference, group B > 40% and group C > 80%, log rank *P* < 0.01.

In further exploratory univariable analysis, those with a VPB of >80% (HR: 1.52, CI: 1.16, 2.03) and a VPB 40–80% (HR: 1.82, CI: 1.44, 2.43) were at increased risk of HFH or all-cause death compared with those with a VPB <40 (*P* < 0.01) (*Figure 7*). Using a binary cut-off, a VPB of >20% (vs. ≤20%) was associated with shorter time to HFH or all-cause of death (HR = 1.64, CI :1.09–2.45; *P* = 0.02), however, in the multivariable analysis, none of the three VPB groups, nor the binary >20% cut-off remained significantly associated with the primary endpoint.

## Discussion

The present study describes several important novel findings. Firstly, we describe the prevalence and clinical characteristics associated with LVSD in patients receiving *de novo* RVP pacemaker therapy and those undergoing replacement in the contemporary era of RVP avoidance programming, and show that baseline LVSD was common, particularly among patients with multiple cardiovascular comorbidities, and especially in those with IHD and a high VPB. Secondly, we demonstrate that patients undergoing *de novo* implantation have a higher rate of LVSD than those undergoing a replacement, yet those undergoing a generator replacement have a higher rate of HFH and mortality. Finally, we demonstrate that VPB is not an isolated driver of adverse clinical outcomes, and that comorbidities including older age, IHD and AF, as well as the number of co-morbidities present, could form part of a more comprehensive risk assessment. Although only a minority of patients in real-world practice develop a clinically significant reduction in LVEF after 12 months, our findings suggest that such events are concentrated in more vulnerable individuals. Together, these observations could help identify a population in whom targeted screening, optimization of medical therapy, and tailored pacing strategies could be clinically beneficial and an effective use of resource to reduce patient-oriented adverse events.

### The adverse effect of RVP

Given the heterogeneity of definitions of pacing-induced cardiomyopathy (PICM) and its typically delayed presentation, the present findings are best interpreted as describing pacing-associated left ventricular systolic dysfunction (LVSD) rather than definitive PICM. LVSD represents a clinically relevant diagnostic marker and predictor of adverse outcomes and has been reported to occur more frequently in people with RVP for bradycardia compared to the general population.^26^ Although more severe LV dysfunction defined as LVEF ≤35% has traditionally been used to guide CRT implantation in heart failure populations, thresholds for considering CRT in patients undergoing pacing for bradycardia with an anticipated high RVP burden are less well defined and vary across guidance and studies. Recommendations from the ESC and Heart Rhythm Society support consideration of CRT at higher LVEF ranges (approximately 40–50%) to mitigate pacing-associated LVSD,^27,28^ consistent with evidence from studies such as the BLOCK-HF trial.^29^ In contrast, studies of CRT upgrade, including the BUDAPEST-CRT Upgrade trial,^25^ have been conducted in patients receiving bradycardia pacing who have developed LV dysfunction (LVEF ≤35%) and should be interpreted as addressing a distinct clinical scenario rather than informing LVEF thresholds for *de novo* CRT implantation. This threshold reflects treatment eligibility rather than the biological onset of systolic impairment. We therefore selected an LVEF cut-off of <50% to capture earlier stages of LVSD, where mechanical dyssynchrony may already be present and clinically relevant. This approach is supported by contemporary HF guidelines, which acknowledge historical changes in nomenclature and the lack of consensus regarding the optimal LVEF threshold to define LVSD in patients without overtly reduced EF.^15^ The Candesartan in Heart Failure: Assessment of Reduction in Mortality and Morbidity (CHARM) programme originally introduced the concept of ‘preserved’ EF to describe patients with an LVEF >40% that was neither clearly reduced nor entirely normal, while current ESC guidelines classify patients with an LVEF of 41–49% as having heart failure with mildly reduced ejection fraction, although whether this group is a distinct clinical phenotype with a meaningful difference in morbidity and risk of disease progression is unclear.^30–32^ In addition, the EACVI defines LVSD using sex-specific thresholds (<52% for males and <54% for females),^15,33,34^ further supporting the clinical relevance of systolic impairment at LVEF values above conventional CRT thresholds.

The association between long-term ventricular pacing, LVSD and HF-related events has led to the development of algorithms encouraging intrinsic rhythm whenever possible.^35,36^ Historical device trials, such as the Mode Selection Trial in Sinus-Node Dysfunction (MOST)^37^ and the Dual Chamber and VVI Implantable Defibrillator (DAVID),^38^ demonstrated that a high burden of RVP was associated with adverse outcomes, including an increased risk of HFH and AF. However, in the MOST study, which investigated patients with sinus node disease and a normal QRS duration, participants did not undergo systematic echocardiography, limiting the ability to define causation. Consequently, it remains unclear if the conclusions drawn from MOST are applicable to all pacemaker recipients, especially those who fall outside the trial’s inclusion criteria, and those receiving medical and device treatments that could affect the incidence or progression of LVSD.^36^

Whilst MOST^37^ and DAVID^38^ described that those with a VPB beyond 40% were more likely to experience HF-related events, the results of the Effect of Biventricular Upgrade on Left Ventricular Reverse Remodelling and Clinical Outcomes in Patient With Left Ventricular Dysfunction and Intermittent or Permanent Apical/Septal Right Ventricular Pacing (BUDAPEST-CRT Upgrade)^25^ randomized trial (*n* = 360) highlighted the potential benefit of CRT in addition to an implantable cardioverter defibrillator on clinical outcomes, in people with VPB of only >20%. In our study, RVP alone at levels >80% was associated with adverse outcomes; however, lower rates were not independently associated with outcomes, hence, considering pacing burden as an isolated driver may be less informative than evaluating it in combination with other clinical and imaging factors.

### Contribution of comorbidities

Baseline LVSD was common in our cohort, particularly among patients with multiple cardiovascular comorbidities. A history of IHD was strongly associated with prevalent LVSD, probably reflecting underlying myocardial fibrosis and mechanical dyssynchrony.^39,40^ LVEF also decreased progressively with increasing numbers of comorbidities, suggesting that LV vulnerability is largely driven by underlying cardiac disease rather than VPB alone. The association of LVEF <40% with a lower risk of HFH and death initially appears counterintuitive, given that a lower LVEF is generally associated with worst outcomes. This paradoxical finding can be attributed by several possible explanations. The lower comorbidity score observed in the generator replacement group may partly reflect a survivorship effect; patients with multiple comorbidities are less likely to survive to undergo a generator replacement. Patients with severely reduced LVEF may have been treated more comprehensively, including optimized HF medication management and device therapy, which could have reduced their risk of HFH and death despite their low baseline LVEF. This is supported by observed treatment intensification often seen in patients with severe heart failure.^32,41^ Moreover, the multivariable analysis adjusted for clinically relevant variables, showing that LVEF <40% is associated with an increased risk of HFH and death, further strengthens the hypothesis that low LVEF is indeed a risk factor when considering cofounding factors. Interestingly, VPB > 80% was no longer a significant predictor in adjusted model, indicating that the effect of high VPB might be cofounded by other factors, such as comorbidities and treatment strategies, which are not captured by the univariable analysis alone. Thus, while LVEF <40% was initially associated with a lower risk of HFH and death, the multivariable analysis suggest that this effect could be due to more aggressive treatment and patient selection factors, which highlight the complexity of the relationship between LVEF, pacing burden and clinical outcomes. Further studies, including sensitivity analyses and more granular data on treatment modalities, are needed to better understand this complex interaction. These findings highlight the importance of identifying patients with pre-existing LVSD prior to pacemaker implantation.

### Identifying those at risk of incident LVSD

Among patients undergoing RVP, those with evidence of focal fibrosis on cardiovascular magnetic resonance (CMR) prior to implantation experienced greater deterioration in LV function compared with patients without fibrosis.^42^ CMR therefore provides strong discriminatory value in identifying patients at risk of adverse remodelling, with fibrosis predicting progression to severe LVSD in around one-fifth of patients, outcomes that include eligibility for CRT upgrade and potential HFH. However, routine CMR screening is impractical in real-world practice, as patients requiring pacing frequently present with advanced conduction disease or profound bradycardia acutely. In this context, simple clinical variables provide a pragmatic alternative for risk stratification. In our cohort, a history of IHD and a requirement of VPB >80% were associated with more than a twofold increased risk of developing LVSD, while being older, having history of AF, and lower baseline LVEF independently predicted HFH or death. Among these, AF is particularly notable, as it often precedes HF and identifies a high-risk population,^43^ particularly in those with a high VPB.

Most patients maintained their LV function following *de novo* RVP, but in the small proportion who experienced a clinically significant reduction in LVEF, VPB was not usually an isolated driver of progression. Patients requiring higher VPB often also had several cardiac comorbidities, suggesting that progression of LVSD and adverse events may reflect the underlying cardiac disease that necessitated pacing,^44–46^ rather than pacing burden alone. In practice, a strategy in which simple clinical variables guide risk stratification could help to identify patients who may benefit from closer monitoring or alternative pacing strategies, especially when advanced imaging such as CMR is not feasible.

### Mitigating the risk of RVP: optimized programming

Whilst VPB might not be the only driver of adverse clinical events, reducing VPB is likely to remain important, not least for battery longevity. The present study showed that patients undergoing *de novo* demonstrated a markedly lower overall requirement for pacing compared to those at replacement, both in terms of atrial and ventricular pacing burden. This probably reflects differences in underlying conduction system disease and device indication, with a higher proportion of *de novo* patients maintaining intact atrioventricular conduction. Such patients may benefit most from pacing minimization strategies, as their intrinsic rhythm can be preserved with appropriate programming. In addition, though approximately 30% of patients implanted with a pacemaker with documented AVB actually have intact conduction at their first outpatient assessment,^47^ hence, programming to permit intrinsic conduction is essential. Furthermore, simply personalizing programming to reduce unnecessary RVP can improve LV systolic function and indexed LV end systolic volume within 6 months, with no detrimental effect on quality of life.^21^ RVP avoidance algorithms are therefore recommended,^48^ and should be widely adopted to extend longevity and improve clinical outcomes.^49,50^

Managing patients with unavoidable RVP due to complete and persistent AVB remains challenging. Comprehensive management strategies, including optimized device programming, medical therapy, alternative RV lead position or upgrade to CRT,^6^ should be considered to improve clinical outcomes in these patients.

### Primary prevention of HF

The traditional approach of apical RVP has been applied for more than half a century due to its proven safety and reliability,^1,2,51^ yet it is widely agreed that RVP can exacerbate the progression of LVSD in at-risk populations.^52^ Alternative pacing sites have not been universally successful at mitigating this risk.^53,54^ CSP attempts to achieve more physiological ventricular activation, preserving ventricular synchrony, haemodynamic function,^55^ myocardial work,^56^ and quality of life,^57^ and although observational studies hint at benefits on clinical outcomes, issues of non-randomized study designs leading to heterogeneity across patient groups limit their relevance.^58^ Moreover, success rates and efficacy in the real world remain unclear. For example, HBP has an 80% success rate even in experienced hands, and routinely requires higher pacing outputs, possibly underlying its lack of wide adoption.^14,59–61^ On the other hand, left bundle branch area pacing (LBBAP) has a higher success rate, shorter procedure and fluoroscopy time, lower pacing thresholds and higher R-wave amplitudes than HBP and has become more favoured, justifying further randomized trials.^62^ The results of the ongoing double-blind Physiological vs. Right Ventricular Pacing Outcome Trial Evaluated for Bradycardia Treatment (PROTECT-HF) (*n* = 2600), comparing traditional apical (or septal) RVP and CSP (LBBAP and HBP) on patient-orientated endpoints will help determine whether CSP should be routine, or reserved for those at greatest risk.^63^ If the hypothesis is proven correct, patient identification for physiological pacing referral will be key.

The current study provides features that could identify a group of patients with RVP that could benefit from alternative pacing strategies, specifically, those predicted to have higher VPB requirements, a history of IHD, and AF. From a patient-orientated perspective, our observed event rate of 11% will also aid calculation of sample size requirements to show clinical benefit of pacing therapies compared with a real-world cohort of patients with contemporary pacemaker programming.^26,64^

## Limitations

Our data demonstrate important findings, but some limitations should be acknowledged. Firstly, these data reflect single centre experience, albeit from a representative and large volume centre. The differences in recruitment intervals between *de novo* and replacement groups pose a significant limitation in the interpretation of findings. As patients were recruited from different years, variations in the treatment guidelines, and pacing programming practices could have influenced the outcomes. For instance, the replacement group had already received a mean of 8 years of pacemaker therapy and also seemed to be taking less optimal medical therapy. The pacing programming itself may have evolved with newer technologies and clinician practices, which might not be comparable between groups. Similarly, pharmacological advancements or changes in the standard of care over the years could have impacted the management of patients in the study. Additionally, there was an insufficient echocardiographic dataset available for the PGR group at follow-up. As a result, changes EF from baseline could only be assessed in the *de novo* group, limiting comparative analysis. These temporal variations in clinical management represent an inherent cofounding factor that may impact the comparability of the two groups. As such, caution must be exercised when drawing conclusion based on these two groups, as the difference in treatment approaches may not be fully accounted for in the analysis. Further research, ideally with more uniform recruitment periods and standardized protocols, would be needed to validate and strengthen the finding from this study. Secondly, clinical and biomarker data were restricted by pre-specified study protocols, limiting the ability to describe the value of NT-pro BNP, for example, in predicting adverse outcomes in people with or receiving pacemakers. Thirdly, there is likely to be some survival bias in the replacement group, leading to underrepresentation of certain high-risk individuals. This potential survival bias should be considered when interpreting differences in baseline comorbidity between *de novo* and replacement cohorts. To address this, stratified analyses could be beneficial in longer-term studies. Additionally, although patients were stratified based on their ventricular pacing burden (<20%, 40–80%, and >80%), the pacing status within the study introduces potential bias. Variations in patients being assessed in either intrinsic or paced rhythm may influence cardiac function and haemodynamic, thereby reducing the validity of comparing groups within the cohort. Future studies should focus on patients requiring high degree of right ventricular pacing, which will allow for better standardization of the cohort and reduce this inherent bias. Furthermore, this study provides observational data, and thereby causation cannot be established.

Finally, it is also possible that the effect of RVP on outcomes was underestimated, as follow-up may have been insufficient to capture events that occur later, cumulative pacing burden over time may be more relevant than single time-point measurements.

In addition, inclusion bias must be considered. A proportion of patients with RVP and LVSD may, under contemporary guidelines, have been eligible for CRT at the time of implantation or generator replacement. Therefore, this cohort likely represents a selected real-world population in whom LV dysfunction was either unrecognized, not reassessed contemporaneously, or not treated with CRT. This limits generalizability to current standards of care and may reflect historical variability in screening practices, referral pathways, and device selection strategies.

## Conclusions

This study highlights the complexity of clinical decision-making with regards to providing optimal device prescriptions for people requiring pacemaker therapy for bradycardia. Our findings suggests that cardiovascular comorbidities in conjunction with ventricular pacing requirements, are associated with the prevalence and development of LVSD and adverse clinical outcomes. These observations may help inform risk stratification and the design of clinical trial cohorts, facilitating focussed exploration of novel protective pacing delivery, device programming and pharmacology strategies.

## Supplementary Material

oeag069_Supplementary_Data

## Data Availability

Individual participant data that underlie the results reported in this article will be available after de-identification (text, tables, figures, and appendices) beginning 9 months and ending 36 months after article publication. Investigators requesting access will require a methodologically sound proposal approved by an independent review committee identified for this purpose to achieve the aims in their approved proposal. Data will be provided within 3 months of a request. Proposals should be directed to the corresponding author (M.F.P.).
