# Supplementary material for: Prevalence and incidence of left ventricular systolic dysfunction and adverse outcomes in patients receiving de novo and replacement pacemaker therapy for bradycardia
Source: Eur Heart J Open. 2026 Apr 27;6(3):oeag069. doi: 10.1093/ehjopen/oeag069 (PMC13252588; doi:10.1093/ehjopen/oeag069)
Supplement: oeag069_Supplementary_Data [file oeag069_supplementary_data.docx]

**Table S1 Factors associated with left ventricular systolic dysfunction**

| **Variables** | **Parameter**  **Estimate** | **Standard**  **Error** | **Wald x^2^**  **P-Value** | **Odds Ratio**  **(95% CI)** |
| --- | --- | --- | --- | --- |
| **(A) Unadjusted odds ratios** |  |  |  |  |
|  |  |  |  |  |
| **Patient Group (replacement)** | 0.10 | 0.14 | 0.15 | 1.21 (0.93 – 1.57) |
| **Sex (male)** | 0.65 | 0.15 | <0.01* | 2.19 (1.66 – 2.91) |
| **Age < 75 (years)** |  |  |  | 1.00 (ref) |
| **75 – 80** | 0.28 | 0.27 | 0.29 | 1.33 (0.78 – 2.24) |
| **>80** | 0.49 | 0.23 | 0.03* | 1.64 (1.04 – 2.57) |
| **IHD** | 0.70 | 0.16 | <0.01* | 2.49 (1.80 – 3.44) |
| **Type II Diabetes Mellitus** | 0.01 | 0.19 | 0.54 | 1.12 (0.76 – 1.62) |
| **AF** | 0.40 | 0.15 | 0.03* | 1.36 (1.03 – 1.81) |
| **VPB <40 (%)** |  |  |  | 1.00 (ref) |
| **40 – 80** | 0.85 | 0.21 | 0.11 | 1.72 (0.89 – 3.34) |
| **>80** | 0.54 | 0.34 | <0.01* | 2.34 (1.55 – 3.52) |
|  |  |  |  |  |
| **(B) Adjusted odds ratios** |  |  |  |  |
|  |  |  |  |  |
| **Patient Group (replacement)** | -0.11 | 0.27 | 0.83 | 0.94 (0.56 – 1.59) |
| **Sex (Male)** | 0.40 | 0.29 | 0.07 | 1.67 (0.95 – 2.93) |
| **Age < 75 (years)** |  |  |  | 1.00 (ref) |
| **75 – 80** | 0.06 | 0.33 | 0.78 | 1.09 (0.58 – 2.05) |
| **>80** | 0.27 | 0.28 | 0.26 | 1.37 (0.79 – 2.35) |
| **IHD** | 0.933 | 0.25 | <0.01* | 2.56 (1.58 – 4.13) |
| **Type II Diabetes Mellitus** | 0.02 | 0.29 | 0.47 | 1.22 (0.71 – 2.09) |
| **AF** | 0.24 | 0.26 | 0.09 | 1.52 (0.93 – 2.51) |
| **VPB <40 (%)** |  |  |  | 1.00 (ref) |
| **40 – 80** | 0.06 | 0.44 | 0.88 | 1.06 (0.45 – 2.49) |
| **>80** | 0.76 | 0.26 | 0.01* | 2.13 (1.29 – 3.52) |
| Values are expressed as odds ratio (95% Confidence Interval). * Denotes as statistical significance p < 0.05.  IHD; Ischaemic Heart Disease, AF; atrial fibrillation, APB; atrial pacing burden, VPB; ventricular pacing burden | | | | |

**Table S2 Factors associated with event free to first outcome of all-cause mortality or HFH**

| **Variable** | **Parameter**  **Estimate** | **Standard**  **Error** | **Wald x^2^**  **P-Value** | **Hazard Ratio (95% CI)** |
| --- | --- | --- | --- | --- |
| **(A) Unadjusted hazard ratios** |  |  |  |  |
|  |  |  |  |  |
| **Patient Group (replacement)** | 1.11 | 0.13 | 0.02* | 3.03 (2.35 – 3.96) |
| **Sex (Male)** | 0.16 | 0.11 | 0.91 | 1.18 (0.90 – 1.55) |
| **Age <75 (years)** |  |  |  | 1.00 (ref) |
| **75 - 80** | 0.21 | 0.31 | 0.48 | 1.24 (0.68 – 2.26) |
| **>80** | 0.88 | 0.24 | <0.01* | 2.41 (1.51 – 3.84) |
| **IHD** | 0.01 | 0.16 | 0.98 | 1.00 (0.73 – 1.37) |
| **Type II Diabetes Mellitus** | 0.10 | 0.19 | 0.60 | 1.11 (0.76 – 1.61) |
| **AF** | 0.81 | 0.14 | <0.01* | 2.25 (1.73 – 2.94) |
| **VPB <40 (%)** |  |  |  | 1.00 (ref) |
| **40 – 80** | 0.27 | 0.22 | 0.21 | 1.32 (0.85 – 2.04) |
| **>80** | 0.76 | 0.29 | 0.01* | 2.14 (1.21 – 3.76) |
| **LVEF >50 (%)** |  |  |  | 1.00 (ref) |
| **40 – 50** | -0.13 | 0.28 | 0.65 | 0.88 (0.51 – 1.53) |
| **<40** | -0.70 | 0.29 | 0.02* | 0.50 (0.28 – 0.87) |
| **LVESD (mm)** | 0.01 | 0.01 | 0.54 | 1.01 (0.99 – 1.03) |
| **LVEDD (mm)** | 0.01 | 0.01 | 0.57 | 1.01 (0.99 – 1.03) |
|  |  |  |  |  |
| **(B) Adjusted hazard ratios** |  |  |  |  |
|  |  |  |  |  |
| **Patient group (replacement)** | -2.49 | 1.01 | 0.02* | 2.27 (1.78 – 4.16) |
| **Sex (male)** | -0.29 | 0.29 | 0.35 | 0.76 (0.43 – 1.35) |
| **Age <75 (years)** |  |  |  | 1.00 (ref) |
| **75 - 80** | 0.28 | 0.37 | 0.45 | 1.32 (0.64 – 2.72) |
| **>80** | 0.78 | 0.31 | 0.01* | 2.19 (1.19 – 4.05) |
| **IHD** | 0.03 | 0.27 | 0.68 | 1.11 (0.68 – 1.82) |
| **Type II Diabetes Mellitus** | -0.02 | 0.30 | 0.94 | 1.03 (0.60 – 1.75) |
| **AF** | 0.88 | 0.26 | 0.01* | 2.26 (1.37 – 3.72) |
| **VPB <40 (%)** |  |  |  | 1.00 (ref) |
| **40 – 80** | 0.25 | 0.35 | 0.79 | 1.08 (0.61 – 1.89) |
| **>80** | 0.07 | 0.28 | 0.47 | 1.29 (0.65 – 2.54) |
| **LVEF >50 (%)** |  |  |  | 1.00 (ref) |
| **40 – 50** | 0.49 | 0.41 | 0.23 | 1.64 (0.73 – 3.68) |
| **<40** | 0.57 | 0.26 | 0.04* | 1.77 (1.30 – 3.03) |
| **LVESD (mm)** | 0.02 | 0.03 | 0.54 | 1.02 (0.96 – 1.08) |
| **LVEDD (mm)** | -0.02 | 0.03 | 0.47 | 0.98 (0.93 – 1.04) |
| Values are expressed as hazard ratio (95% Confidence Interval). * Denotes as statistical significance p < 0.05.  IHD; Ischaemic Heart Disease, AF; atrial fibrillation, VPB; ventricular pacing burden, LVEF; left ventricular ejection fraction, LVESD; left ventricular end-systolic diameter, LVEDD; left ventricular end-diastolic diameter. | | | | |
